# Mental Health Symptoms and Service Use in Depressed and Anxious Minors at the Onset of COVID-19 in a County Clinic Serving a Predominantly Hispanic Population

**DOI:** 10.7759/cureus.55160

**Published:** 2024-02-28

**Authors:** Nandhini Madhanagopal, Ammar Ahmad, Yu-Hsi Hu, Garth Olango, Mohammed Molla

**Affiliations:** 1 Psychiatry, Child and Adolescent Psychiatry, Kern Medical, Kern Behavioral Health & Recovery Services, UCLA-Kern Child & Adolescent Psychiatry Fellowship Program, Bakersfield, USA; 2 Psychiatry, Child and Adolescent Psychiatry, Veterans Administration, Palo Alto, USA

**Keywords:** worsening psychiatric symptoms, early covid-19, anxiety, depression children and adolescents, mental health service use

## Abstract

Objective: The study's primary aim was to compare the utilization rates of services by minors with depression/anxiety in a county mental health clinic before (from December 1, 2019, to March 15, 2020) and during the COVID-19 pandemic (from March 16 to June 30, 2020). The secondary aim was to study demographics and psychiatric symptomatology.

Methods: Service utilization rates were estimated. Univariate and multivariate logistic regression was used to identify significant predictors of worsening psychiatric symptoms, anxiety, and change in the frequency of therapy between the pre-COVID-19 period and the COVID-19 period.

Results: Service utilization rates increased during the pandemic period. During the pandemic, the presence of mood symptoms, suicidal ideation, and relationship conflicts predicted worsening psychiatric symptoms. In addition, the presence of preexisting sleep problems and physical health issues that continued during COVID-19 exhibited correlations with worsening psychiatric symptoms during COVID-19. COVID-related stressors and physical health issues were associated with anxiety; suicidal ideation predicted a change in the frequency of therapy.

Conclusions: Prospective studies to recognize risk factors for worsening mental health in minors with psychiatric illness during a crisis are warranted to identify and allocate services to the high-risk groups.

## Introduction

Although children and adolescents were less afflicted by COVID-19, they are one of the groups most impacted by the social changes implemented to prevent the pandemic’s spread [1]. Worsening mental health and educational gaps are becoming more obvious, and enormous interventions may be necessary to address these concerns. Many studies have reported elevated depressive symptoms by one in four youths and elevated anxiety symptoms by one in five youths in the first year of the pandemic [2]. Studies have also reported a steep surge in telehealth services when shelter-in-place orders were implemented during the COVID-19 pandemic. In California, a shelter-in-place order was issued on March 19, 2020, leading to a rapid transition from in-person to 100% telehealth services in our child and adolescent mental health outpatient clinic in Kern County, CA, serving a predominantly Hispanic population.

Despite the high rates of mental health challenges, Hispanic youths have low rates of mental health care utilization [3]. To our knowledge, this is the first study that examines the symptomatology and service utilization rates of children and adolescents with pre-existing depression and anxiety early in the pandemic in a predominantly Hispanic population. Mental health service utilization in the early pandemic has been variable. One study noted that youths were more likely to terminate mental health services during the first month of the pandemic when in-person services were moved to telehealth [4]. A study in Canada documented that mental health visits were low from March (the onset of the pandemic in Canada) to June 2020; however, the visits increased from July 2020 onward for adolescent females. The authors attributed the increase in outpatient mental health care visits to the onset of the second COVID wave in Ontario in the fall of 2020 and the continued rise in the winter of 2020 and early 2021 at the peak of lockdown measures [5].

Children with preexisting mental health conditions had higher levels of parent-reported mental health symptoms in an Australian study [6]. Pediatric patients with psychiatric disorders reported poorer socioemotional and school functioning [7], and stressful life events are one of the factors that impact their quality of life and may interfere with recovery from internalizing problems [8]. Susana et al. described female gender, lack of control over the situation, dissatisfaction with measures to address COVID-19, and reduction in interaction with family and friends enhanced the worsening of preexisting psychiatric conditions in adults during the pandemic [9]. Mental health practitioners strive to provide the best care for their patients and being aware of factors that increase the risk of worsening psychiatric symptoms during a large-scale disaster such as the COVID-19 pandemic may assist practitioners in treatment planning while they triage available finite resources.

In this study, our main objective was to measure the changes in mental health service utilization during the early stages of the COVID-19 pandemic by comparing the service utilization rates three and a half months before and after the onset of the COVID-19 pandemic. As a secondary objective, we sought to characterize the changes in the presentation of minors to mental health appointments (therapy and/or medication management visits) prior to and during the pandemic. We hypothesized that mental health service utilization rates will increase during the COVID-19 pandemic associated with a transition from in-person to virtual visits. Furthermore, building on existing literature suggesting an increase in anxiety, depression, and other psychiatric symptoms during the pandemic [9], we also hypothesized that minors with preexisting depression and anxiety may experience exacerbated symptoms during this period.

## Materials and methods

We conducted a retrospective chart review of child and adolescent patients with a preexisting diagnosis of depression and/or anxiety who attended one of the psychiatry outpatient clinics at the Kern Behavioral and Health Recovery System (KBHRS). The KBHRS is contracted through the State of California as the managed care entity for Medi-Cal beneficiaries in Kern County. The Institutional Review Board at Kern Medical approved this study protocol, and the IRB waived informed consent as this was a retrospective chart review.

The study period was December 1, 2019, to June 30, 2020. The period from December 1, 2019, to March 15, 2020, was identified as the pre-COVID-19 period, and the period from March 16 to June 30, 2020 is the period during COVID-19. The State of California declared a state of emergency on March 4, 2020, and the Kern County public schools were temporarily closed on March 15, 2020. KBHRS clinics closed for in-person services and moved to virtual services on March 16, 2020. Patients continued seeing their therapist and psychiatrist virtually, and phone visits and video visits were conducted, with a preference for video visits.

**Figure 1:**
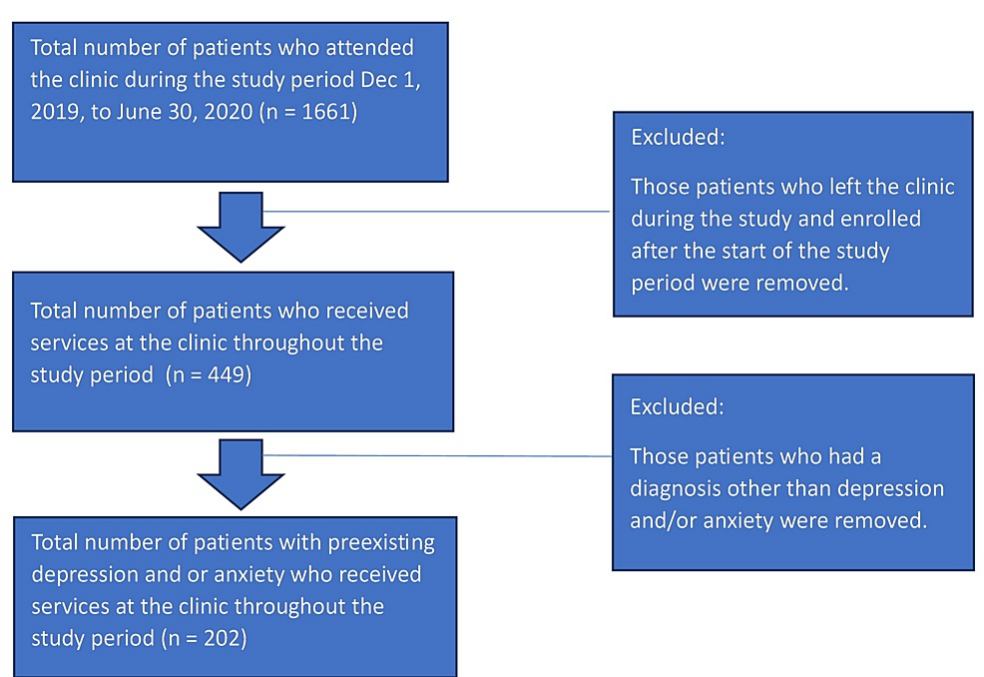
Selection process for the retrospective chart review

Patients who attended the clinic from December 1, 2019, to June 30, 2020, and had a diagnosis of depression and/or anxiety were included in the study, and those who did not receive services for this period and those who did not carry a diagnosis of depression or anxiety were excluded. A total of 449 children and adolescents who attended the clinic during the entire study period were considered for study enrollment. Restricting the participation of individuals with preexisting depression and/or anxiety yielded a final count of 202 participants. Figure 1 shows the details. 

Study protocol

Patient demographics, including age, gender, ethnicity, language preferred by the patient and the caregiver, residence, legal guardianship status, highest level of education, and use of educational supports, such as 504 plans or Individualized Education Programs (IEPs), were collected. Parameters of interest included psychosocial stressors, presenting symptoms, psychiatric diagnoses, and treatment received by the patient. 

A data abstraction instrument was created using an Excel sheet with columns representing the above categories parallel to the information flow in the health record, and a drop-down menu with “yes” and “no” options was created and distributed. Two meetings were held with the residents collecting data to train them on data abstraction methods. A pilot test was conducted after collecting data on 10% of the targeted sample to ensure that the data abstractors understood the process and to evaluate the appropriateness of the data abstraction instrument.

Statistical analysis

Mental Health Service Utilization

Mental health service utilization in this study refers to the use of mental health services at the county clinic by the study population. We reported the mental health service utilization by using the number of people utilizing mental health services in the clinic in the respective study period (December 1, 2019, to March 15, 2020, was identified as the pre-COVID-19 period and March 16 to June 30, 2020, as during the COVID-19 period) divided by the current population of Bakersfield as the denominator [10]. The mental health service utilization rate was presented as per 100,000 population. Percentage differences were calculated to identify changes in the above two periods.

Psychiatric Symptoms

The collected data were divided into four different categories: psychosocial stressors, presenting symptoms, psychiatric Dx, and treatment. All data, except for those in the "COVID-related stressors in the psychosocial stressors" category, contained pre- and post-COVID measurements. All variables are bivariate except for the number of therapy visits attended, number of therapy visits missed, number of med visits attended, and number of med visits missed in the "Treatment" category of variables.

All pre- and post-COVID bivariate data were examined using an exact McNemar’s test (based on the binomial distribution) for dependent proportions. This allowed us to look specifically at pre-COVID versus post-COVID responses individually variable by variable.

Three predictor variables were selected for logistic regression analyses to enhance the clinic's ability to pinpoint patients necessitating a higher level of care, thus facilitating a refinement of the emergency preparedness protocol. These variables include (1) worsening psych symptoms, (2) anxiety, and (3) change in frequency of therapy, aligning with the clinic's imperative to optimize patient management and resource allocation.

Three different univariate logistic regression analyses were conducted using each of the above three variables as the dependent variables. The same process was used for all the dependent variables. Univariate logistic regression was used to predict each of the identified dependent variables. Those that showed mild statistical significance (p-value ≤ 0.10) were included in the initial multivariate model. A combination of forward and backward elimination was used to reduce the model to a more parsimonious model consisting only of predictor variables with statistical significance (p-value ≤ 0.05) in the presence of all other statistically significant predictors. Odds ratios were used to address the strength of the effect size.

## Results

Demographic characteristics

The study participants included 202 children and adolescents with preexisting depression and/or anxiety, with 113 (55.94%) females, 84 males (41.58%), and five biological females wishing to transition to the male gender (2.48%). The predominant ethnicity in the sample was Hispanic 126 (63.32%), followed by non-Hispanic Caucasian (36, 18.09%) and mixed (32, 16.08%). Full demographics are depicted in Table 1.

**Table 1:** Demographic characteristics of the study population IEP: Individualized Education Program

Characteristic	N	%	Mean	SD
Age			14.10	2.85
Gender				
Female	113	55.94		
Male	84	41.58		
Transgender (F to M)	5	2.48		
Ethnicity				
Hispanic	126	63.32		
Caucasian non-Hispanic	36	18.09		
Mixed	32	16.08		
Others	5	2.51		
Education				
Elementary school	31	15.35		
Middle school	40	19.80		
High school	130	64.36		
College	1			
Caregiver language				
English	141	70.5		
Spanish	58	29.0		
Other	1	0.5		
Patient language				
English	198	98.02		
Spanish	2	0.99		
Other	2	0.99		
Residence				
Home	190	94.06		
Foster home	10	4.95		
Other	2	0.01		
Guardianship				
Biological parents	146	73.74		
Other relatives	26	13.13		
Adoptive parents	14	7.07		
Ward of the court	12	6.06		
504 or IEP at school				
Yes	82	26.84		
No	115	73.16		

Changes in outpatient mental health service utilization

Mental health service utilization increased from pre-COVID-19 (40.23 per 100,000 population) to during COVID-19 (48.58 per 100,000 population) and was accompanied by a rapid switch from in-person to virtual care at the onset of the COVID-19 pandemic. This corresponded to a remarkable 20.7557% increase in mental health service utilization by minors with pre-existing depression and/or anxiety.

Mental health at the onset of the COVID-19 pandemic

A logistic regression was used to determine risk factors associated with the worsening of psychiatric symptoms, presentation with anxiety, and change in frequency of therapy during the COVID-19 pandemic (Table 2). The predictor variables were chosen based on p-values, and the Akaike information criterion (AIC) was used to set a limit on the total number of variables.

**Table 2:** Predictors of worsening psychiatric symptoms, anxiety, and change in the frequency of therapy OR: odds ratio, CI: confidence interval

Parameter	Variable	Coefficient	OR	95% CI	p-value
Worsening psychiatric symptoms (n = 156)	Mood	2.5863	13.2802	(2.5254, 69.8352)	0.0023
	Conflict with girl/ boyfriend	3.0981	22.1558	(3.3781, 145.3111)	0.0012
	Suicidal ideation	4.8912	133.1155	(12.7278, 1392.2083)	<0.0001
	COVID-19-related stressors	1.5376	4.6533	(1.3721, 15.7811)	0.0136
	Physical health issue	2.4783	11.9210	(1.1309, 125.6626)	0.0391
	Sleep	1.6540	5.2276	(1.3905, 19.6527)	0.0144
Anxiety (n = 81)	Physical health issue	2.5281	12.5292	(1.2300, 127.6226)	0.0328
	COVID-19-related stressors	1.1049	3.0189	(0.9619, 9.4746)	0.0583
Change in frequency of therapy (n = 193)	Suicidal ideation	2.2007	9.0315	(2.0547, 39.6974)	0.0036

Predictors of Worsening Psychiatric Symptoms During COVID-19

When restricting the dataset to those without worsening psychiatric symptoms pre-COVID-19, the number of patients was reduced from 202 to 156.

Issues with girlfriend/boyfriend: Individuals reporting conflict with their girlfriend or boyfriend during COVID-19 are approximately 22.1558 times more likely to experience worsening psychiatric symptoms compared to those not reporting issues in their relationships with their girlfriend or boyfriend (OR = 22.1558, p = 0.0012). 

Suicidal ideation: Individuals reporting suicidal ideation during COVID-19 are approximately 133.1155 times more likely to experience worsening psychiatric symptoms compared to those not reporting suicidal ideation (OR = 133.1155, p < 0.0001).

Mood: Individuals reporting mood symptoms during COVID-19 are approximately 13.2802 times more likely to experience worsening psychiatric symptoms compared to those not reporting mood symptoms (OR = 13.2802, p = 0.0023).

COVID-19-related stressors: Individuals reporting COVID-19-related stressors during COVID-19 are approximately 4.6533 times more likely to experience worsening psychiatric symptoms compared to those not reporting COVID-19-related stressors (OR = 4.6533, p = 0.0136).

Physical health issues: Individuals reporting physical health issues before and during COVID-19 are approximately 11.9210 times more likely to experience worsening psychiatric symptoms compared to those not reporting physical health issues (OR = 11.9210, p = 0.0391).

Sleep issues: Individuals reporting sleep issues before and during COVID-19 are approximately 5.2276 times more likely to experience worsening psychiatric symptoms compared to those not reporting sleep issues (OR = 5.2276, p = 0.0144)

Predictors of Anxiety

When restricting the dataset to those not reporting anxiety pre-COVID-19, the number of patients was reduced from 202 to 81. One hundred patients reported anxiety pre-COVID-19 and during COVID-19, and these patients were excluded from the analysis.

Physical health: Individuals reporting physical health issues during COVID-19 are approximately 12.5292 times more likely to report anxiety during COVID-19 compared to those not reporting physical health issues (OR = 12.5292, p = 0.0328).

COVID-19-related stressors: Individuals reporting COVID-19-related stressors during COVID-19 are approximately 3.0189 times more likely to report anxiety during COVID-19 compared to those not reporting COVID-19-related stressors (OR = 3.0189, p = 0.0583).

Predictors of Change in Frequency of Therapy

When restricting the dataset to those without a change in the frequency of therapy pre-COVID-19, the number of patients was reduced from 202 to 193.

Suicidal ideation: Individuals reporting suicidal ideation during COVID-19 are approximately 9.0315 times more likely to report a change in the frequency of therapy during COVID-19 compared to those not reporting suicidal ideation (OR = 9.0315, p = 0.0036).

## Discussion

Mental health service utilization

Our study demonstrated a 20.7% increase in the utilization of mental health services by children and adolescents with preexisting depression and/or anxiety in the first three months of the COVID-19 pandemic compared to the three months before the onset of the pandemic. The increase in service use by our study population could be attributed to the presence of existing telehealth infrastructure before COVID-19 to provide mental health services for rural communities and to the availability of tablets that were free of cost for students to allow virtual classes by the public schools.

Saunders et al. noted an initial decrease in physician-based outpatient mental health visits for the first four months and then an increase of 10-15% above expected for the first 12 months of the pandemic [5]. Burrell et al. [11] did not document the initial drop in mental health visits described by Saunders et al. [5] or an increase in mental health visits due to the stress of the pandemic. Thus, mental health service utilization by children and adolescents during the COVID-19 pandemic has been heterogeneous. Studies examining trends across various demographics, such as age, gender, race, and psychiatric diagnosis, are essential for understanding the nuanced complexities involved.

Virtual mental health visits emerged as the predominant mode of outpatient mental health care in the literature [5,11], as evidenced by all outpatient appointments in our sample transitioning to virtual visits during the pandemic. This shift highlights the potential of virtual care as a promising solution to enhance the timeliness of patient care and improve patient satisfaction. Though the first few sessions were offered by telephone, the preference for video format sessions by our clinic could also have impacted the increased service use by youth in our study [12].

Barriers to accessing telehealth services by marginalized populations are well-established [13]. Burrell et al. observed that psychopharmaceutical prescribing occurred predominately for Whites compared to non-Whites during the pandemic [11]. Nevertheless, our study documented an increase in mental health service utilization by the minoritized population it serves during the pandemic. Qualitative and longitudinal studies must be conducted among the minoritized population to improve our understanding of the role of individual and societal level barriers to equitable access to mental health care.

Predictors of Worsening Symptoms, Anxiety, and Increase in the Frequency of Therapy

The presence of suicidal ideation, mood symptoms, sleep problems, physical health issues, COVID-19-related stressors, and conflicts in relationships with a girlfriend or boyfriend predicted the worsening of psychiatric symptoms in this study as depicted in Table 2. The presence of COVID-19-related stressors and physical health issues were associated with a presentation of anxiety during the COVID-19 period and the presence of suicidal ideation predicted a change in the frequency of therapy during the pandemic.

An umbrella review by Hossain et al. [14] reported an increased prevalence of anxiety, depression, sleep disorders, suicidal behavior, stress-related behaviors, and other psychosocial challenges among children and adolescents in the setting of epidemiological isolation during the COVID-19 pandemic. Our study shared some of the findings of this review. The presence of suicidal ideation and mood symptoms during the COVID-19 study period predicted the worsening of psychiatric symptoms in this study. Worsening depression and anxiety in youth during COVID-19 is well documented in the literature; however, suicidal ideation was noted to have worsened after the summer of 2020 with an initial stabilization during the early phase of the pandemic [15]. This study extends the existing association between worsening depression and anxiety and increased suicidal ideation [16]; however, it noted worsening depression and anxiety during the first three months of the pandemic unlike the initial stabilization reported in the above study, thus highlighting the importance of close follow-up with minors with pre-existing mental illness during periods of widespread disasters, such as COVID-19.

Post-relationship concerns, particularly breakups, demonstrated the most pronounced correlations with mental health issues, suicide, and self-harm risk [17]. Our study mirrored this finding, indicating a significant association between conflicts with romantic partners during the COVID-19 study period and the exacerbation of psychiatric symptoms. Relationships beyond family play a vital role in the social development of adolescents and relationship loss was identified as a risk factor in first-onset depression [18].

Those who reported physical health issues and sleep problems during both the pre-COVID-19 and COVID-19 study periods also reported worsening psychiatric symptoms. This result aligns with the report by Kiss et al. that pre-pandemic sleep problems contributed to worsening distress during COVID-19 [19]. The literature on the impact of large-scale disasters, such as the COVID-19 pandemic on adolescents with both mental and physical health problems, is limited. Aarons et al. have described that adolescents with both mental and physical health problems are likely to have a low quality of life in adulthood, and mental health interventions have been shown to improve physical health outcomes in this population [20].

In this study, minors who reported physical health issues only during the COVID-19 study period and COVID-19-related stressors, such as missing graduation and inability to socialize with peers, were associated with a presentation of anxiety, whereas Panchal et al. [21] identified special needs, preexisting mental health disorders, and excessive screen exposure as risk factors for anxiety during the pandemic. Latinos overall and Latino children specifically were disproportionally represented in both the COVID-19 infection and mortality rates in California [22]. A study [23] highlighted that presenting with symptoms at a health clinic for COVID-19, as well as hospitalization due to the virus, along with exposure to family members with COVID, exacerbated anxiety among children and adolescents. Given our study's focus on a predominantly Hispanic population, it is plausible that similar factors contributed to the study population’s manifestation of anxiety during the pandemic.

The presence of suicidal ideation during the COVID-19 study period is associated with a change in the frequency of therapy. Suicidal ideations can be regarded as an indicator of the severity of psychiatric illness in this population, which will warrant an escalation in mental health services. This finding was in line with the reported increase in demand for therapy for the treatment of anxiety and depression reported by psychologists in an American Psychological Association (APA) survey in 2021 [24]. Saunders et al. conducted a population-based study and noted that physician-based outpatient mental health visits declined for the first four months after the onset of COVID-19, followed by a swift increase in visits 10-15% above expected through the rest of the first year of the pandemic [5].

Strengths

Our research possesses several notable strengths. It significantly adds to the essential body of knowledge concerning mental health utilization rates among minors experiencing depression and/or anxiety during the early stages of the COVID-19 pandemic - a period marked by the collective challenge of adapting to social limitations imposed by quarantine measures. The findings of our study offer valuable insights into the symptomatology observed in depressed and/or anxious children and adolescents when confronted with significant disasters, such as the COVID-19 pandemic. Furthermore, our study identifies associations with worsening psychiatric symptoms, the manifestation of anxiety, and changes in the frequency of therapy. These identified associations serve as crucial avenues for further exploration in prospective studies. By delving into these aspects, future research can deepen our understanding of the nuanced dynamics involved in the mental health of minors during times of crisis. It is noteworthy that our study population predominantly comprises individuals of Hispanic descent, making a significant contribution to the literature on minoritized populations. This inclusion is essential for fostering a more comprehensive and inclusive understanding of mental health dynamics within diverse communities.

Limitations

While our study yields valuable insights, it is essential to acknowledge its inherent limitations. The data, derived from a retrospective chart review, may carry potential inaccuracies stemming from incomplete data entry. It is noteworthy, however, that such errors are likely evenly distributed across pre-pandemic and pandemic periods, minimizing their impact on the overall study outcomes. In addition, our study was conducted in a clinic serving a predominantly Hispanic population, and caution is to be exercised in applying these results to other minoritized populations. Another limitation is that the study was conducted in a county clinic where most patients are on Medi-Cal. The results of this study may not be generalizable to the uninsured or those with private insurance. Furthermore, the study examined data immediately before and after the start of the COVID-19 lockdown, and the results may not be generalizable to the long-term effects of the pandemic. Despite these constraints, the study uncovers compelling observations that bear significance for addressing the mental health of children and adolescents. These insights can inform resource allocation and strategic planning in future pandemics, underscoring the study's contribution to the broader discourse on public health resilience.

## Conclusions

The results of this study indicated an increase in mental health service rates early in the pandemic by minors with pre-existing depression/anxiety, accompanied by a rapid shift to virtual services. This points toward the need for planning and implementation to meet the increased need and provide timely services during a crisis period. Although telehealth has been promising in increasing access and patient and provider satisfaction, the quality and impact of virtual services in the long term should be examined further.

The predictors of worsening mental health, anxiety, and a change in the frequency of therapy identified in this study point to the need for multisectoral support for minors with pre-existing mental illness during a crisis involving mental health providers, teachers, and parents. This preparedness could be achieved by educating minors, parents, and teachers about symptoms for which they should seek mental health support. Further prospective studies that identify risk factors for worsening mental health in minors during a crisis are warranted to identify and allocate services to the high-risk groups.
